# Image Stabilization in Central Vision Loss: The Horizontal Vestibulo-Ocular Reflex

**DOI:** 10.3390/vision2020019

**Published:** 2018-04-13

**Authors:** Esther G. González, Runjie Shi, Luminita Tarita-Nistor, Efrem D. Mandelcorn, Mark S. Mandelcorn, Martin J. Steinbach

**Affiliations:** 1 Krembil Research Institute, Toronto Western Hospital, Toronto, ON M5T 2S8, Canada; 2 Department of Ophthalmology and Vision Sciences, University of Toronto, Toronto, ON M5T 2S8, Canada; 3 Centre for Vision Research, York University, Toronto, ON M3J 1P3, Canada; 4 Department of Biomedical Engineering, University of Toronto, Toronto, ON M5T 2S8, Canada

**Keywords:** vestibulo-ocular reflex, eye movements, age-related macular degeneration, central vision loss, preferred retinal locus

## Abstract

For patients with central vision loss and controls with normal vision, we examined the horizontal vestibulo-ocular reflex (VOR) in complete darkness and in the light when enhanced by vision (VVOR). We expected that the visual-vestibular interaction during VVOR would produce an asymmetry in the gain due to the location of the preferred retinal locus (PRL) of the patients. In the dark, we hypothesized that the VOR would not be affected by the loss of central vision. Nine patients (ages 67 to 92 years) and 17 controls (ages 16 to 81 years) were tested in 10-s active VVOR and VOR procedures at a constant frequency of 0.5 Hz while their eyes and head movements were recorded with a video-based binocular eye tracker. We computed the gain by analyzing the eye and head peak velocities produced during the intervals between saccades. In the light and in darkness, a significant proportion of patients showed larger leftward than rightward peak velocities, consistent with a PRL to the left of the scotoma. No asymmetries were found for the controls. These data support the notion that, after central vision loss, the preferred retinal locus (PRL) in eccentric vision becomes the centre of visual direction, even in the dark.

## 1. Introduction

In developed countries where infection and nutritional/metabolic deficits are no longer major causes of visual impairment, age-related macular degeneration (AMD) is the leading cause of legal blindness in people 65 years and older [1]. This progressive disease, characterized by the dysfunction and eventual death of the macula’s photoreceptors, can culminate in the loss of central vision [2,3].

Central vision loss results in deficits in visual functions such as acuity [4], contrast sensitivity [5], colour discrimination [6,7], insensitivity to flicker [8,9], delayed dark adaptation [10], shape perception [11], face recognition [12,13], stereopsis [14] and reading [15,16]. Patients adapt to the loss of central vision with the habitual use of an area of eccentric retina called the preferred retinal locus (PRL) as the new point of reference for the ocular motor system [17,18,19] Research on the ocular motor consequences of central vision loss has included fixation stability [20,21], saccades [22,23,24], smooth pursuit [25,26,27,28] and optokinetic nystagmus [29]. The vestibulo-ocular reflex (VOR) of patients with AMD and other low vision patients with various aetiologies has been explored along with other predictors of the functional success of telescopic spectacle use [30,31]. The objective of the present paper, however, is to examine the characteristics of the VOR of patients with central vision loss with a particular interest in the effects of their eccentric viewing, that is of their PRL.

The VOR, also known as the oculocephalic reflex, minimizes the motion of a target on the retina during head movement by producing compensatory eye movements in the direction opposite to those of the head, thus preserving the image in the center of the visual field and reducing retinal slip. Movements of the head can be either active (e.g., fixating straight ahead while rotating the head) or passive (e.g., fixating straight ahead while sitting on a rotating chair or when the head is moved by an experimenter or clinician). The VOR occurs after about 8 ms, and this short latency implies that during head movement when the eyes fixate a stationary target, the required adjustments occur too fast to be visually guided. Furthermore, the VOR also occurs in the dark. The direct VOR arc involves the vestibular nerve, the vestibular nuclei and their projections to the ocular motor nuclei. The cerebellum can modulate the VOR through its connection with the vestibular nuclei [32,33]. The cervico-ocular reflex also generates smooth eye movements in response to neck movements, but its magnitude is small in healthy humans [33] and, in contrast to the VOR, is most responsive at low velocities [34].

The gain of the VOR is the ratio of eye to head velocity. When the movements of the eyes have the same speed as the rotational movements of the head, the image of the visual target is stabilized on the person’s retina, and the gain of the VOR value is one [35] (compensatory eye movements that are in the opposite direction of head motion are conventionally assigned a positive gain). In the light, vision enhances the gain of the reflex (VVOR) by using the optokinetic response at very low frequencies (~0.1 Hz) and smooth pursuit eye movements and the fixation system at higher frequencies (≥2 Hz). Lower VOR gain values of 0.6 to 0.9 are achieved in darkness during passive head rotations in adults, but at very high frequencies of head motion, the VVOR is independent of fixation and smooth pursuit [36]. Both cancellation and enhancement of the VOR can occur in the absence of a visual fixation target if the subject imagines a head-fixed or Earth-fixed target during vestibular stimulation in the dark [37,38,39].

We examined angular VVOR and VOR performance during active, i.e., volitional, horizontal head motion. We tested patients with central vision loss and controls with normal vision in light and in darkness in order to evaluate the effects of eccentric viewing on this reflex. Our prediction was that the location of the PRL would produce asymmetrical VVOR gains if the PRL were on either the left or the right side of the scotoma in the visual field. For rightward eye movements and a PRL on the left of the scotoma, a low gain would eventually bring the image of the visual target into the area of the scotoma while for leftward eye movements, the target’s image would remain in an area of healthy vision. The converse would be true for a right PRL, but PRLs above or below the scotoma should produce no asymmetries. In the absence of visual feedback, the VOR of the patients, however, should exhibit no asymmetries.

In daily life, VOR responses typically occur at frequencies higher than 1 Hz [40], but in this exploratory study, we used a slow frequency of 0.5 Hz in order to prevent dizziness or discomfort in our elderly patients. Furthermore, the elderly tend to have difficulties producing higher frequency movements of the head [41].

## 2. Methods

This research was approved by the University Health Network’s Research Ethics Board and conducted in accordance with the tenets of the Declaration of Helsinki. Patients were recruited from the Eye Clinic at the Toronto Western Hospital, and controls were students, volunteers or hospital staff. Written informed consent was obtained from all individual participants included in the study.

### 2.1. Participants

Demographic and clinical characteristics of all participants are shown in Table 1 and Table 2. The same group of patients and most of the controls also participated in a smooth pursuit test that was given after a short break [25].

#### 2.1.1. AMD Group

This group, described in Table 1, was comprised of 9 patients with a confirmed diagnosis of bilateral AMD (based on an optical coherence tomography of the macula or fluorescein angiography) ranging from early to late according to the Beckman Initiative Classification [42]. They all had no other neurological or ocular pathologies with the exception of mild cataracts (2+ or less in a four-point grading system modified from the Lens Opacities Classification System II (LOCS II) [43]) and were under treatment for neovascular AMD or scheduled for visits to monitor disease progression for intermediate AMD. Their ages ranged from 67 to 92 years (mean = 78.9, SD = 8.8). Coincidentally, the right eye was the eye with the better acuity for all patients.

#### 2.1.2. Control Group

All 17 controls had normal or corrected to normal visual acuity and a stereopsis score of at least 40 arc s^−1^. Because of the difficulties inherent in finding age-matched controls without any ocular pathologies, we decided to include a control group with a wide age range from 16 to 81 years (mean = 40.35, SD = 21.72). The right eye of these participants was used in analyses with the exception of control participant C17 (Table 2) who reported diplopia during testing, but not during calibration. Analysis of the eye position records showed that the left eye was closer than the right eye to the 0,0 position of the calibration target. This person’s left eye was therefore used in all analyses, but the differences between the two eyes are shown for illustrative purposes.

### 2.2. Equipment

Binocular horizontal and vertical eye positions were recorded using an integrated system consisting of a head-mounted video-based infrared eye tracker (Series 2020; El-Mar, Inc., Toronto, ON, Canada) and a magnetic head tracking system (Flock of Birds; Ascension Technology Corp., Shelburne, VT, USA) [44]. The eye-tracker has a sampling rate of 120 Hz, is free of drift and has a maximum resolution of 6 min arc. Its linear range is at least 30 deg in the horizontal and 25 deg in the vertical meridians. This equipment’s accuracy and precision are comparable to those of magnetic search coil technique [45].

Head position was recorded using a single receiver mounted on a frame fitted on top of the participants’ head at about the centre of yaw rotation of the head. This receiver senses a pulsed magnetic field transmitted from an Earth-fixed unit at a rate of 60 Hz. Head position (x, y, z) and orientation (azimuth, elevation, roll) are recorded in 3 dimensions with a noise of 0.1° RMS and a 180° linear range, approximately within a 1-m^3^ space [44]. The distance from the centre of the head rotation to the centre of the eyes’ rotation in the sagittal plane was about 10 cm, depending on the dimensions of the participant’s head.

The calibration targets and fixation stimulus were generated using VPixx, a graphics and psychophysical testing software (VPixx Technologies, Inc., Montreal, QC, Canada). Each calibration target was a nine-cycle black and white square-wave radial grating subtending 3 deg. For patients with AMD, this type of target has been shown to produce fixation stability that is largely independent of visual acuity [46]. The fixation target was a 1 deg white dot (122 cd/m^2^) presented on a black background (2.7 cd/m^2^).

Stimuli were presented at a viewing distance of 57 cm on a Samsung monitor (Sync Master 900 NF; Samsung, Seoul, Korea) with an effective field of view of 34.4 × 26 cm, a resolution of 1024 × 768 pixels and a refresh rate of 120 Hz. Participants were seated in an expressly designed chair that brings the eyes of people with healthy retinas and normal binocular vision into primary position when looking at the center of a computer screen. During calibration, the head was steadied by a chinrest. Our eye tracker does not allow the use of corrective spectacles, and the viewing distance of 57 cm ensured that the target was visible for all the participants.

### 2.3. Procedure

It has been reported that the habitual use of corrective spectacles affects VOR gain [47,48], so participants were instructed to remove their optical correction one hour before testing.

After the eye movement recordings were over, visual acuity was obtained with a computerized version of the Early Treatment of Diabetic Retinopathy Study (ETDRS) test. This test was performed at 6 m using a letter-by-letter scoring method with the participants wearing their habitual distance spectacles. Stereoscopic acuity was measured using the Fly Stereotest (available in the public domain at http://www.stereooptical.com).

For the patients only, a fundus photograph and a 15-s eye fixation recording with the MP-1 microperimeter (Nidek Technologies Srl., Vigonza, Padova, Italy) were performed monocularly for each eye. Fixation stability was measured with a bivariate contour ellipse area (BCEA) and the location of the PRL relative to the vestigial fovea (Table 3) defined as its centroid. The distance between the PRL and the former fovea was estimated using the MP-1′s built-in grid using average values for a normal population [21].

The chinrest was removed after the eye tracker’s software declared the calibration successful, which, in the case of the patients, meant that they used the same PRL for viewing the centre of each one of the 14 targets. The test of the VVOR followed. Participants were instructed to fixate on the central 1 deg target while moving their head side-to-side about 15 deg from the centre of the screen (i.e., close to the edge of the field of view of the monitor). They were given one or more practice trials and tested after it was clear that they understood the task. Each trial started with a fixation task lasting 15 s, after which the computer provided a 1-s warning tone followed 3 s later by 20 tones of a 0.1-s duration that marked each half cycle of head motion (in other words, the tones were produced at a rate of 1 Hz making the VOR’s frequency 0.5 Hz.). The trial ended with another 15 s of target fixation without head movement.

The VVOR trial was done under standard fluorescent lighting. For the VOR test, which always followed the one in the light, participants saw the visual target before it was covered with a thick black cloth and, after the lights were turned off, had to remember its location and keep “fixating” on it. Care was taken that no stray lights were visible in the room.

### 2.4. Data Analysis

**Figure 1:**
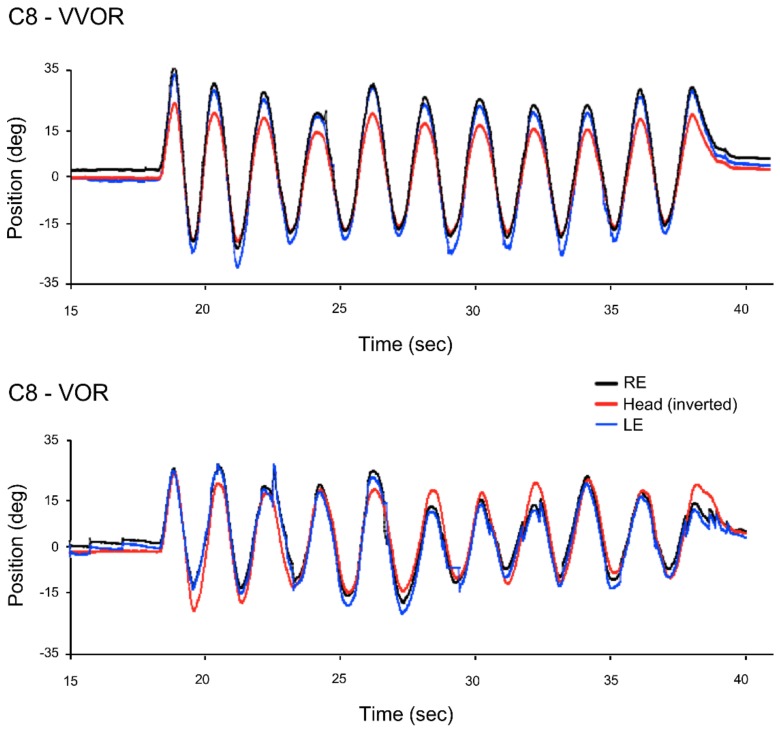
Right (RE), left (LE) eye and head position traces in the light (VVOR, above) and in the dark (vestibulo-ocular reflex (VOR), below) for the complete testing period for C8, one of the control participants. The values of the head’s position coordinates are inverted so that the eye and head curves can be overlapped.

**Figure 2:**
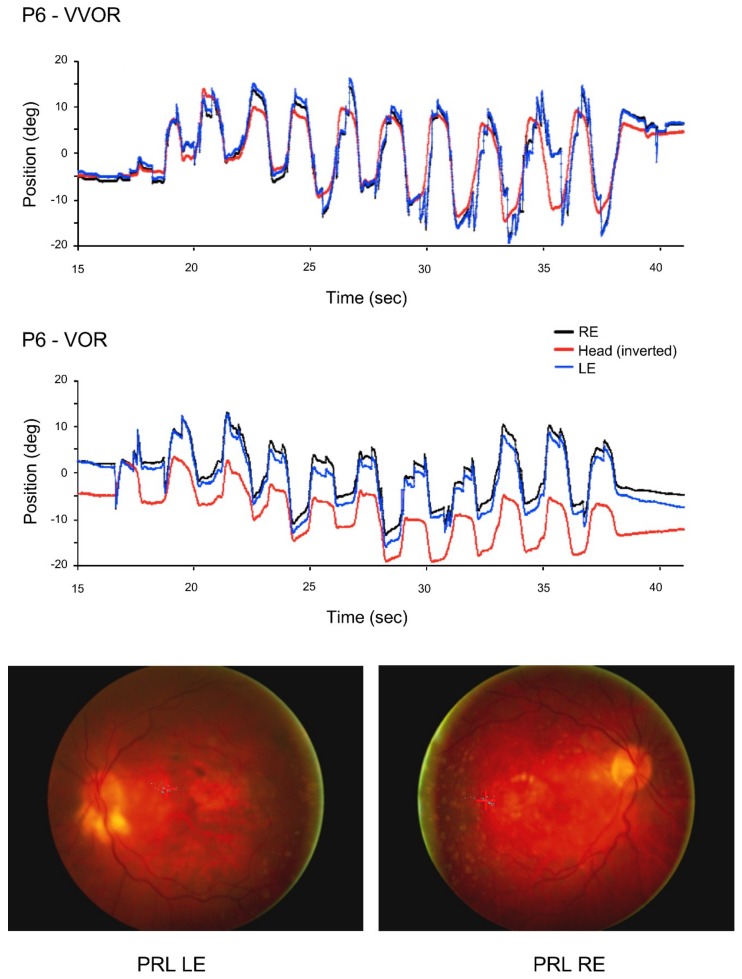
For patient P6, right (RE), left (LE) eye and head position traces in the light (VVOR, above) and in the dark (VOR, below) for the complete testing period. The values of the head’s position coordinates are inverted so that the eye and head curves can be overlapped. Fundus photographs show that the preferred retinal locus (PRL) is located on the left of the scotoma in the visual field for both eyes.

All the patients and most of the control participants had never been involved in an eye movement recording experiment, and although the frequency of their head movement was fairly accurate, its amplitude was not (Figure 1 and Figure 2). Patients in particular produced large and frequent saccades.

Because we were especially interested in analyzing the asymmetry of the eye movements of the patients, we computed the VVOR and VOR gains by analyzing the eye and head peak velocities produced during the intervals between saccades. This method allowed us to analyze cycles characterized by non-periodicity and asymmetry. Gain was computed as the ratio of the peak velocity of the eye and the head movements. We also measured the kinematics of the saccades produced.

### 2.5. Gain

The usual measure of VOR is its gain defined as the angular velocity of the eye divided by the angular velocity of the head measured in the same plane. For targets nearer than optical infinity, an offset between the centers of head and eye rotation results in a VOR gain larger than unity because the centre of rotation of the head is behind that of the eyes and these have to move faster than the head in order to avoid slippage of retinal images. In 1987, Hine and Thorn published an equation based on the head rotation angle and the following measures: interocular distance, viewing distance and the distance between the centre of the head and the centre of rotation of one of the eyes [49]. We used a simpler approximation of the expected gain of either eye (*G_R/L_*) of the form,

(1) $$G_{R/L}=\frac{\left(x+h\right)}{x}$$

where *x* is the viewing distance (57 cm) and *h* the distance between the centre of rotation of the head to the cyclopean centre of rotation of the eyes in the frontal plane (10 cm). Using average head sizes, this method produces a similar expected gain of 1.17.

To calculate the eye and head’s peak velocity, custom software written in Matlab™ displayed the eye and head position data allowing the operator to select the epochs between saccades to be analyzed. For each epoch, but separately for the eye and head signals, the software performed a numerical first-order differentiation with 4 neighbouring data points using the five-point stencil technique. The data were separated in terms of whether the eye movement was toward the better (right) eye or against it.

### 2.6. Saccades

Saccadic amplitude and velocity were calculated after differentiating eye position data with respect to time. Using a 3-point differentiator, custom software written in Matlab™ defined saccades as eye movements with a minimum start velocity of 50 deg/s. It displayed the differentiated signal under the eye position data and marked the beginning and end of each saccade. Using the mouse and guided by the differentiated signal, the operator could delete spurious saccades or mark missed ones. Saccades were classified as forward or regressive. Only saccades with a magnitude greater than 0.1 deg were included.

The alpha level was set at 0.05 for all the statistical tests, and when required, a Greenhouse–Geisser correction for violations of the sphericity assumption was applied.

## 3. Results

Sample eye and head position traces of a control participant (Figure 1) and a patient (Figure 2) during the 10 s of VOR recording were the result.

### 3.1. Gain

**Figure 3:**
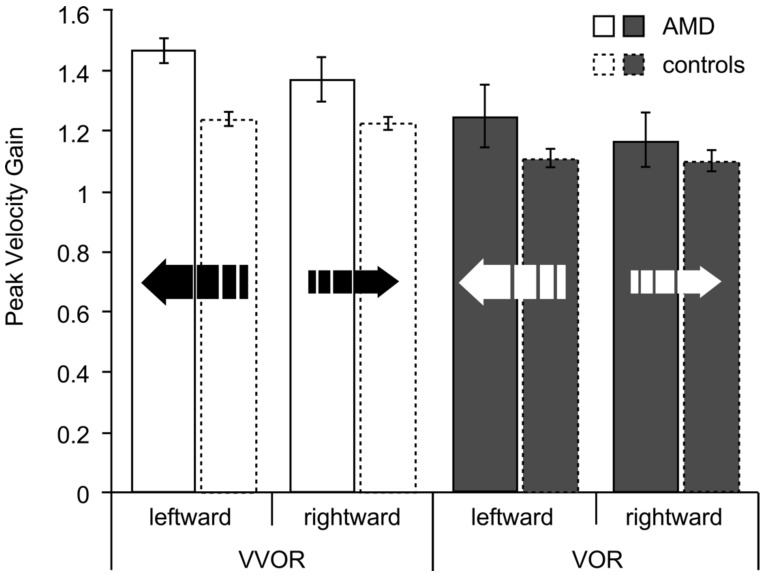
For the AMD (solid lines) and control groups (dashed lines), VVOR and VOR gain derived from the peak velocity of leftward and rightward eye movements between saccades. Error bars are ±1 SE.

For the AMD group, leftward eye movements exhibited a higher gain than rightward eye movements: for the VVOR, 78% (seven out of nine) of the patients exhibited a higher gain for leftward movement, and for the VOR 89% (eight out of nine) showed this preponderance. For the controls, the preponderances of leftward gain values were 47% and 53% for VVOR and VOR, respectively. The data are shown in Figure 3.

**Figure 4:**
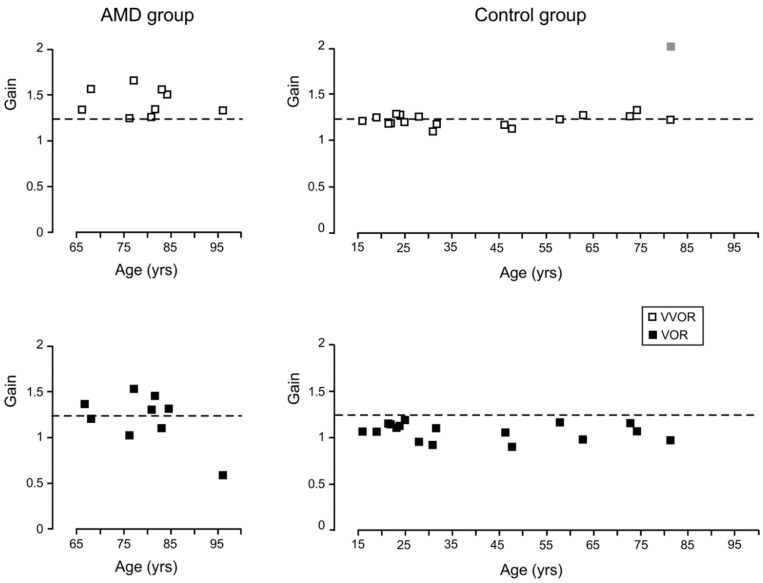
Mean gain values (average of rightward and leftward eye movements) obtained from the eye’s peak velocity between saccades. All the data were obtained from the participants’ right eye with the exception of control participant C17 who reported diplopia and whose left eye datum is shown as a black open square and the right eye’s as a grey filled square. For all the other participants in both the control and AMD groups, there were no significant differences in gain between the two eyes. The dashed lines show the expected average gain of 1.17 (Equation (1)).

The peak velocity gain was analyzed with a 2 × 2 × 2 ANOVA for groups (AMD, control), direction (leftward, rightward) and lighting condition (VVOR, VOR). This analysis yielded a significant effect of group, F(1,24) = 7.00, *p* = 0.01, partial η^2^ = 0.23, direction, F(1,24) = 8.17, *p* = 0.009, partial η^2^ = 0.25, lighting condition, F(1,24) = 19.80, *p* < 0.001, partial η^2^ = 0.45, and a significant group × direction interaction, F(1,24) = 5.12, *p* = 0.03, partial η^2^ = 0.18. Analysis of this group × direction interaction showed that the patients had significantly greater gains than the controls only for the leftward gain, t (24) = 3.28, *p* = 0.003. Interestingly, control participant C17, whose left eye was used for analysis, also exhibited a leftward VVOR gain preponderance for the right eye: a gain of 1.85 for rightward eye movements and 2.23 for leftward eye movements (Figure 4).

The mean VVOR gain of the patients was 1.41 (SD = 0.15) and their mean VOR 1.20 (SD = 0.29). For the controls, the mean VVOR was 1.23 (SD = 0.07) and the mean VOR 1.10 (SD = 0.14). A *t*-test of the residuals (absolute deviations of the observations from the treatment mean) showed that the AMD group was significantly more variable than the controls, t (24) = 2.64, *p* = 0.01.

For the VVOR, the expected gain of 1.17 is below the lower limit of the 95% confidence interval of the mean of both groups. For the VOR, the gain was below (and outside) the confidence interval of the AMD group and within, but at the upper limit of the confidence interval of the controls. Differences in head size make these comparisons difficult.

In order to determine whether the direction effects were due to differences in head velocity, the unsigned values of the peak velocity of the head movements were analyzed with a 2 × 2 × 2 ANOVA for groups (AMD, control), direction (leftward, rightward) and lighting condition (VVOR, VOR), which yielded no significant effects of any of the factors or their interactions (Table 4).

For the patients, there was a statistically-significant correlation between the leftward VVOR gain and fixation stability, r(7) = −0.61, *p* = 0.04. No other significant correlations between gain and fixation stability or between gain and the PRL’s distance to the former fovea were found.

The mean visual enhancement of the VOR (gain_VVOR_/gain_VOR_) for the AMD group was 1.17 and for the controls 1.11.

### 3.2. Saccades

**Figure 5:**
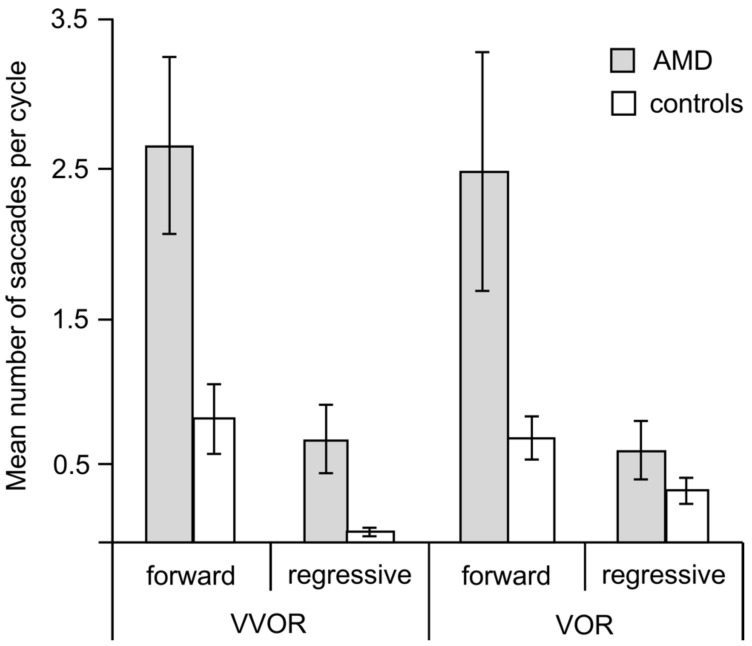
Mean number of saccades per cycle made by the AMD and control groups during VOR testing. Error bars are ±1 SE.

**Figure 6:**
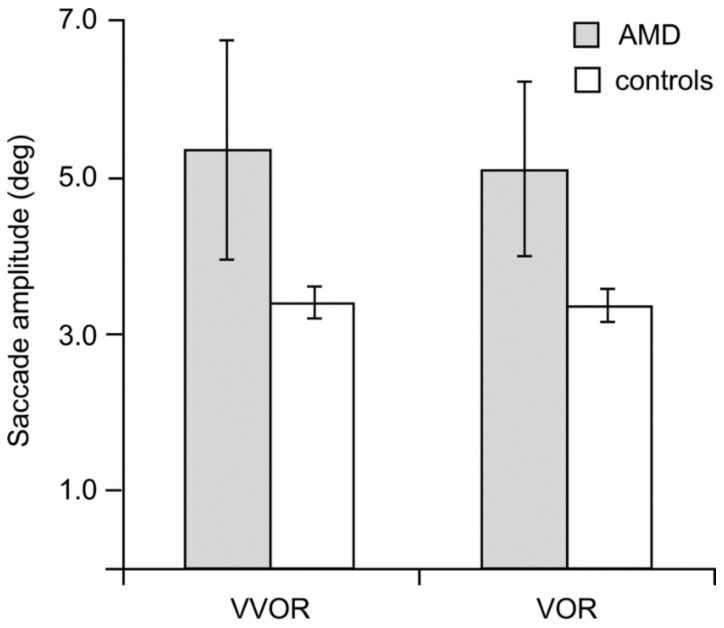
Mean amplitude (in deg) of the forward saccades made by the AMD (N_AMD_ = 9) and control groups (N_control_ = 15) during the 20 s of VOR testing. Error bars are ±1 SE.

We analyzed saccades as forward (catch-up), if in the direction of the eyes’ movements, and regressive, if against them. Figure 5 and Figure 6 show that the patients made more and larger saccades than the controls and that the number of regressive saccades was smaller than that of catch-up saccades for both groups.

A Mann–Whitney U test showed that the patients with AMD made significantly more forward saccades than the controls both during VVOR (z = −2.59, *p* = 0.008) and during VOR (z = −2.10, *p* = 0.03) (Figure 5). Due to their low frequencies, we did not analyze the regressive saccades statistically, but the pattern of the intergroup differences was similar.

The saccadic amplitudes of the AMD group were significantly more variable than the controls as evidenced by Levene’s test of the equality of error variances, which was significant for the analyses of the group (AMD, control), lighting condition (VVOR, VOR) and direction (leftward, rightward) of the catch-up saccades. This prevented parametric statistics from being applied to comparisons between the two groups. Between groups, Mann–Whitney non-parametric tests also failed to reach statistical significance.

Only for the VOR condition, the magnitude of the saccades of the AMD group was significantly correlated with the distance of the PRL to the fovea (Table 1), r(8) = 0.57, *p* = 0.04. Visual acuity correlations with saccadic amplitude, on the other hand, did not reach statistical significance.

### 3.3. Acuity and Age

For the AMD group, there was only a significant correlation between age and the leftward gain of VOR, r(7) = −0.70, *p* = 0.02, but no other correlations with age were significant. For the controls, in the light and in the dark, none of the correlations between age and gain were statistically significant.

None of the correlations between visual acuity and gain were significant for the AMD group, and there was a modest correlation between acuity and fixation stability, r(7) = 0.58, *p* = 0.05.

## 4. Discussion

Patients with AMD exhibit asymmetrical VVOR and VOR gains with a leftward preponderance and produced more and larger saccades than controls with normal central vision. In the light, the larger magnitude and frequency of saccades are expected consequences of the instability of eccentric fixation and of the attempt to move the eyes closer to the target [22], and the leftward preponderance of the left gain during VVOR may be a consequence of eccentric viewing with a PRL to the left of the scotoma. Unexpected findings were the similar asymmetrical gain and the large number and magnitude of saccades made during VOR by the patients.

We hypothesized that, for VVOR, a PRL to the left of the scotoma in the visual field would decrease the rightward gain relative to the leftward gain, while the opposite would be expected for a PRL to the right of the scotoma. This is because, with a left PRL, rightward eye movements and a low gain would eventually bring the target’s image into the area of the scotoma, while for leftward motion, the image of the target would remain in an area of healthy vision. Larger gains, on the other hand, while helping the visual system maintain the image of the target away from the scotoma, would increase the eccentricity of the PRL. Our data could be interpreted as an attempt by the visual system to avoid an increase in the eccentricity of the PRL used for the task. PRLs above or below the scotoma should produce no gain asymmetries.

High gains in the elderly have been reported within the head movement frequency bands of 3 and 4 Hz, and this VOR dysfunction is attributed to inadequate cerebellar control of the magnitude of the compensatory eye movements [41]. The much lower frequency of head movement used in the present study (0.5 Hz) makes this explanation of the high leftward gain of the VVOR and VOR in the AMD group unlikely. Furthermore, the control participants whose ages overlap the age range of the patients (C15 to C17) did not exhibit larger gains than the younger control participants. Although cerebellar dysfunction in the patients cannot be ruled out completely, a more likely source of the gain asymmetry and large magnitude of the compensatory eye movements of the patients is their eccentric viewing.

Calibration of the VOR occurs throughout the lifetime and depends on retinal motion signals during movements of the head [50]. The congenitally blind, for instance, lack VOR, whereas the adventitiously blind retain a VOR response with a reduced amplitude and time constant [51]. In the case of people with central vision loss, if the scotoma is to be avoided, in order to maintain the image of the target in the visual field, the eyes must move faster in the direction of the PRL and slower in the opposite direction. Our data show that the effect of eccentric viewing of the patients is present both for VVOR and VOR. Because the periphery exhibits higher velocity thresholds than central retina [52,53], patients with central vision loss may experience a less accurate calibration of the VOR, which would explain the variability of their responses.

In the dark, in order to produce the VOR, the oculomotor system makes use of a fast, non-visual estimate of the current target location relative to the head using inputs from the otolith organs and the semicircular canals [32]. In the absence of central vision, the estimate of the target’s location relative to the head could be either the patient’s original egocentric location or the PRL. In the present study, the asymmetry of the VOR and the number and magnitude of saccades in the dark are consistent with the idea that the PRL becomes the new centre of visual direction, even in the dark.

The mean magnitude of the preponderance of leftward over rightward eye movements during VVOR and, even more consistently, during VOR is small as would be expected from the fact that the patients exhibit PRLs at different distances from the former fovea. That a significant proportion of patients showed larger leftward than rightward gain differences is consistent with a PRL to the left of the scotoma, which, along with the inferior part of the visual field, is the most common location for the PRL [21,54,55]. The same group of patients exhibited horizontal smooth pursuit asymmetries consistent with a left PRL [25]. Just as with that smooth pursuit task, in the present study, the PRL determined as the centroid of a static fixation task using the microperimeter (Table 3) was not predictive of the asymmetry of the VVOR or VOR. One major difference between the smooth pursuit and VVOR and VOR performances of these patients is that their leftward gain was significantly higher than that of the controls, whereas in the smooth pursuit task, both gains were lower than those of the controls.

Volitional VVOR and VOR produce some degree of asymmetry in the head movements of even the most careful observers, which is an alternative explanation for the gain asymmetry found in the patients’ data by assuming that rightward head movements (being more common or easier for the patients) might produce a higher gain. We tested the peak velocity of the head, however, and found no significant asymmetries or differences between the two groups in VVOR or VOR.

There are a number of limitations in this study that we should acknowledge. The first is the use of a conventional binocular eye tracker that can only measure relative changes in eye position. The absolute location of the PRL can only be determined using monocular instruments such as a specific kind of scanning laser ophthalmoscopes (SLO) or microperimeters such as the MP-1. Our group developed a method for identifying the location of the two eyes’ PRLs relative to the optic disc, but the system has bandwidth limitations and is not widely available [56].

A second limitation is the small size of our AMD sample and the nature of the scotomas of the patients. Single, absolute, well-defined scotomas similar in size in the two eyes would perhaps be ideal for correlations between the degree of pathology and the outcome measures, but, in spite of these problems, we found consistent patterns that are a useful first step in the analysis of VOR in this patient population.

The anatomical structures responsible for the VOR are present at birth, and this reflex occurs in newborns, but there are important differences in its time constants, and the gain does not reach adult levels until childhood [50]. Because of the difficulties involved in recruiting a perfectly age-matched control group free of ocular pathologies such as glaucoma or advanced cataracts, people with a wide range of ages, but healthy vision were included as controls in this study. Although this is a third limitation of this study, we found that for the two tasks we examined, the age of the controls was not found to be significantly correlated with any of the outcome measures.

Although it is known that advanced age has a detrimental effect on the gain of the VOR, the effects of advanced age and low frequencies in the absence of disease are not very strong or consistent [57,58]. Head autorotation test results have been found to be normal in patients 67 to 75 years of age [57], while other research, using faster head movements, has shown abnormalities in a majority of people (86%) between 74 and 91 years old [41]. In spite of the age differences between our two groups, it is clear that in the present study, the VOR gain is more a function of pathology and other factors rather than of the age of the patients.

## 5. Conclusions

The loss of central vision and the adoption of eccentric viewing has an effect on the vestibulo-ocular reflex, even in the absence of visual feedback. This suggests that the PRL becomes the centre of visual direction, although different PRLs may be used for different tasks by the patients. How this adaptation occurs has important consequences for the rehabilitation of patients and should be the focus of further research.

## Figures and Tables

**Table 1:** Demographic and clinical characteristics of the participants with age-related macular degeneration (AMD).

		Acuity (log MAR)				Stage of AMD (Beckman Initiative Scale)	
ID	Age (Years)	RE	LE	Stereo (arc s^−1^)	Time Since Diagnosis (Years)	RE	LE
P1	67	0.34	1	400	2	late	late
P2	67	0.44	0.86	no stereo	3	early	late
P3	76	0.57	1.2	3600	3	late	intermediate
P4	77	0.24	0.7	3600	1	late	late
P5	81	0.3	0.44	400	1	late	late
P6	81	0.7	2	3600	7	late	late
P7	82	0.4	1	no stereo	3	late	late
P8	83	0.98	1	800	4	late	late
P9	96	0.22	0.64	400	5	early	late

RE = right eye (the better eye for all patients); LE = left eye; inf = inferior; MAR = minimum angle of resolution.

**Table 2:** Demographic characteristics of the control participants.

		Acuity (log MAR)	
ID	Age (Years)	RE	LE
C1	16	0.06	0.04
C2	19	−0.1	−0.1
C3	23	−0.26	−0.28
C4	23	0	−0.1
C5	23	0.06	0.1
C6	24	−0.22	−0.24
C7	25	−0.1	−0.3
C8	28	−0.1	−0.1
C9	31	−0.22	−0.22
C10	31	−0.26	−0.22
C11	46	−0.24	−0.24
C12	48	−0.1	−0.06
C13	58	−0.1	0
C14	63	−0.1	−0.1
C15	73	−0.04	−0.06
C16	74	0.08	0.04
C17	81	−0.1	−0.1

RE = right eye; LE = left eye.

**Table 3:** PRL characteristics of the participants with AMD.

	PRL Location Relative to Scotoma in Visual Field		PRL Distance to Fovea (deg)	Directional Preponderance of Gain		BCEA (deg^2^)	
ID	RE	LE	RE	VVOR	VOR	RE	LE
P1	inferior	left	2	left	left	0.44	0.44
P2	central	inferior	0	left	left	0.16	2.80
P3	inferior	central	3	left	left	1.67	0.46
P4	central	central	0	right	left	1.23	2.76
P5	central	central	3	left	left	0.24	0.12
P6	left	left	7	left	left	2.75	2.34
P7	inferior	left	3	right	left	1.89	1.92
P8	inferior	inferior	4	left	left	1.98	3.16
P9	central	inf/left	2	left	right	0.15	0.94

RE = right eye (the better eye for all patients); LE = left eye; inf = inferior; BCEA = bivariate contour ellipse area.

**Table 4:** Mean peak head velocities (SD) during VVOR and VOR.

	VVOR		VOR	
Group	Rightward	Leftward	Rightward	Leftward
AMD	48.86 (11.25)	47.95 (9.39)	50.81 (12.76)	48.12 (16.75)
Control	58.12 (13.51)	59.65 (14.69)	54.54 (15.98)	55.60 (18.35)
